# Epidemiological Characteristics and Spatiotemporal Patterns of Visceral Leishmaniasis in Xinjiang, China, during 2004–2021

**DOI:** 10.3390/tropicalmed9070153

**Published:** 2024-07-09

**Authors:** Jiangshan Zhao, Yue Zhang, Haiting Zhang, Shuo Wang, Haibo He, Guangzhong Shi, Wumaier Maimaitijiang, Yanyan Hou, Ling Zhang, Jianhai Yin, Yi Wang, Jianping Cao

**Affiliations:** 1 Center for Disease Control and Prevention of Xinjiang Uygur Autonomous Region, Urumqi 830002, China; zjscdc@163.com (J.Z.); zhtcdc@163.com (H.Z.); ws198949@163.com (S.W.); hehaibo17726748835@163.com (H.H.); xjcdcsgz@163.com (G.S.); maimaitijiang2009@126.com (W.M.); woshihouyanyan@163.com (Y.H.); zhangling2613884@163.com (L.Z.); 2 Department of Public Health, Xinjiang Medical University, Urumqi 830000, China; zy2812831338@163.com; 3 National Key Laboratory of Intelligent Tracking and Forecasting for Infectious Diseases, National Institute of Parasitic Diseases at Chinese Center for Disease Control and Prevention, Chinese Center for Tropical Diseases Research, Shanghai 200025, China; yinjh@nipd.chinacdc.cn; 4 Key Laboratory of Parasite and Vector Biology, National Health Commission of the People’s Republic of China, Shanghai 200025, China; 5 World Health Organization Centre for Tropical Diseases, Shanghai 200025, China; 6 The School of Global Health, Chinese Center for Tropical Diseases Research, Shanghai Jiao Tong University School of Medicine, Shanghai 200025, China

**Keywords:** visceral leishmaniasis, spatiotemporal analysis, epidemiological characteristics, autocorrelation analysis, spatial regression

## Abstract

The spread of visceral leishmaniasis (VL), a serious global zoonotic parasitic disease, is mostly under control; however, several cases have been reported in recent decades in Xinjiang, China. This study aimed to analyze the epidemiological status and spatiotemporal clustering characteristics of VL in Xinjiang, China, between 2004 and 2021 to provide a basis for the development and implementation of surveillance and response measures. Data on VL incidence during 2004–2021 were collected from the National Diseases Reporting Information System of China. Global spatial autocorrelation analysis, identification of local indicators of spatial association, and spatial–temporal clustering analysis were conducted to identify the distribution and high-risk areas. A total of 2034 VL cases were reported, with a mean annual incidence of 0.50 per 100,000. There was a general decreasing trend in the incidence of VL during our study period. The majority of the cases were reported from October to February of the following year, and fewer cases were reported from April to July. Spatial autocorrelation analysis revealed that the incidence of VL was spatially clustered within a few counties. Significant differences were observed during the study period (Moran’s I = 0.74, Z = 4.900, *p* < 0.05). The male-to-female ratio was 1.37:1, and most patients were in the age group 0–3 years. Cases were primarily distributed in seven regions and two autonomous prefectures, and Kashgar reported the highest number of cases (1688, 82.98%). Spatial analysis revealed that the aggregation of VL was predominantly observed in southwest Xinjiang. This was in alignment with the high-risk areas identified by spatiotemporal clustering analysis. The H-H clustering region was primarily observed in Gashi, Atushi, Shufu, Injisha, Kashgar, Yepuhu, and Bachu. These findings indicate that integrated control measures must be taken in different endemic areas to strengthen the VL control program in Xinjiang, China.

## 1. Introduction

Visceral leishmaniasis (VL), also known as kala-azar, is a zoonotic parasitic disease caused by a trypanosomatid protozoan of the genus *Leishmania* and transmitted by the bite of infected female sand flies [1]. Its clinical symptoms include intermittent fever, progressive hepatosplenomegaly, anemia, leukopenia, and elevated serum globulin levels, and the mortality rate is over 90% without treatment [2]. It is a severe disease that has become a significant public health issue. Cases of VL have been reported in more than 60 countries all over the world, and this condition is currently prevalent in India, Bangladesh, Sudan, South Sudan, Ethiopia, and Brazil [3]. It was estimated that 0.7–1 million new cases of leishmaniasis were reported annually in nearly 100 endemic countries in 2018 [4]. In recent years, VL has spread and emerged in local areas in China; therefore, it has been classified as a re-emerging parasitic disease by the World Health Organization [5].

In 1904, a confirmed case of VL was reported for the first time in China [6]. In 1951, owing to the lack of effective control measures and inadequate access to treatment, VL was one of the most serious parasitic diseases and was highly prevalent in 16 provinces (autonomous regions and municipalities) north of the Yangtze River in China [7]. Since 1958, after the implementation of nationwide control measures, VL has been controlled and largely eliminated in the northeastern endemic region; however, it is still predominantly endemic in western China, including Kashgar in Xinjiang, as well as in northern Sichuan and southern Gansu [8]. In Xinjiang, VL was first reported by He Guanqing and others in 1948 [9]. After decades of prevention and control, the number of VL cases in Xinjiang decreased sharply, and in 1989, only 11 new cases that were confined to five townships were reported [10]. However, since the mid-1990s, with the ever-increasing population flow, the number of VL cases in Xinjiang has been increasing annually, and they account for 60% of the total number of cases in China [11]. Since 2000, there have been considerable fluctuations in the incidence of VL in Xinjiang. In addition, outbreaks also occurred in Jiashi County and the Kashgar region in 2008 and 2015, respectively, with an incidence rate that was more than 20 times the average annual incidence [12]. Since 2016, VL has been listed in the National Plan for the Control of Major Parasitic Diseases (2016–2020) and its management has been prioritized at the national level. As a result, the spread of VL has been controlled in China, and the number of cases has decreased.

In 1976, Guan [13] classified VL epidemiologically into three types, namely, anthroponotic VL (AVL), mountainous sub-type zoonotic VL (MT-ZVL), and desert sub-type zoonotic VL (DT-ZVL). AVL is caused by *Leishmania donovani*, which is transmitted from human to human, and simultaneous or sequential disease onset is common among family members. MT-ZVL, caused by *L. infantum*, is transmitted through dogs. Its pattern of co-infection between humans and canines is characterized by a low prevalence of patient dispersion. DT-ZVL is caused by *L. infantum* in wild animals [14]. Studies have demonstrated the presence of mainly two types of VL in Xinjiang: AVL and DT-ZVL. While DT-ZVL is mainly distributed in desert and gravelly desert type areas, AVL is primarily distributed in the Kashgar Oasis, which encompasses Kashgar City, Shushu County, Atushi City, and surrounding areas, as well as green agricultural regions in Wushi and Kuqa. Desert-type VL is predominantly found in the desert areas surrounding the Tarim Basin and the Hami Basin, as well as in newly reclaimed desert agricultural areas, including Bachu County, Gashi County, and Korla City. Moreover, gravelly desert-type VL is observed in gravelly Gobi areas located in the foothills and valleys to the north and south of the Tianshan Mountains, which are mainly concentrated in the Turpan and Korla areas [15]. Different types of VL exhibit significant variations in their epidemiological characteristics, including geographic and landscape features, ecosystems, vector species, and sources of infection [14]. Therefore, it is essential to implement targeted measures and allocate limited resources efficiently.

Given the fluctuations in the prevalence of VL, studies on the incidence, variation, and spatiotemporal characteristics of this disease are necessary in Xinjiang. Therefore, this study aimed to investigate the epidemiology of VL in Xinjiang from 2004 to 2021 and assess its spatial and temporal distribution patterns to identify endemic areas and high-risk populations and provide evidence for the development of prevention and control strategies.

## 2. Materials and Methods

### 2.1. Patient Information and Data Collection

VL case data reported in Xinjiang between 2004 and 2021 were obtained from the National Diseases Reporting Information System of China. Population data were obtained from the annual Xinjiang Statistical Yearbook. Counties where VL cases were reported were selected as sampling sites, and the longitudinal and latitudinal coordinates were determined for each site. In the research for the present paper, data on kala-azar cases were extracted from online databases and no human or animal samples were included. All the data analyzed in this study were de-identified to protect patient confidentiality. Therefore, ethical approval and consent to participate were not necessary for the research. All work was carried out in accordance with the relevant guidelines and regulations.

### 2.2. Quality Control

VL cases were diagnosed based on standard WS258—2006 set by the Chinese Ministry of Health [16]. Each VL case record included information on variables such as name, age, gender, diagnosis, date of birth, date of onset, and others. According to the law for the prevention and control of infectious disease, it is mandatory to fill in a standardized infectious disease card for all patients individually. Once the infectious disease card is available, the local epidemiologist conducts a field investigation using a standardized form.

### 2.3. Statistical Analysis

Epidemic data on VL were loaded into Microsoft Excel 2013. Trends related to the VL epidemic were analyzed using a descriptive epidemiology approach, and the data were collated using SPSS (version 23.0). Spatial autocorrelation and cluster analyses were performed using the global Moran’s I and Anselin Local Moran I statistics, respectively, with the ArcGIS software (version 10.8). Global spatial autocorrelation analysis revealed whether clustering or dispersion was present based on the value of Moran’s I. A positive Moran’s I value is considered to indicate the presence of aggregation, whereas a negative value is considered to indicate dispersion [17]. The Anselin Local Moran I statistic was applied for cluster analysis involving the following four patterns: high–high, high–low, low–high, and low–low. A Z-test was used to determine if the differences were significant. The results were visualized using the ArcGIS 10.8. Spatiotemporal aggregation analysis was performed based on the discrete Poisson model at the county and municipal levels, using SaTScan software (version 9.7). The log-likelihood ratio (LLR) and relative risk (RR) of each scan cylinder were calculated with the help of the Poisson Monte Carlo model [18,19]. The difference in RR between the inside and outside of the scan window was considered statistically significant when the *p*-value was less than 0.05. Each scanning window was considered a spatiotemporal cluster [20].

## 3. Results

### 3.1. Trends in Epidemic VL from 2004 to 2021

**Figure 1:**
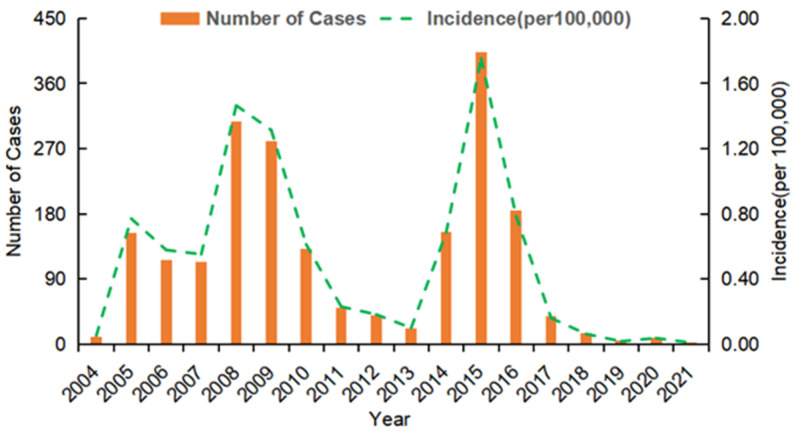
Temporal (yearly) distribution of visceral leishmaniasis (VL) cases (incidence rate) reported in Xinjiang in the period 2004–2021.

A total of 2034 cases of VL were reported in Xinjiang during the years 2004 to 2021. The average annual incidence of VL during this period was 0.50 per 100,000 individuals. The incidence showed an initial increase in 2004 (0.05/100,000), a gradual decline from 2005 to 2007, a dramatic increase and peak in 2008 (1.47/100,000), and then a steady decline until 2013. Subsequently, the trend reversed again and peaked in 2015 (1.75/100,000), after which the incidence decreased each year until 2021. Therefore, a steadily declining trend in the incidence of VL in Xinjiang was evident, although there were marked fluctuations, with outbreak peaks in 2008 and 2015 (Figure 1).

**Figure 2:**
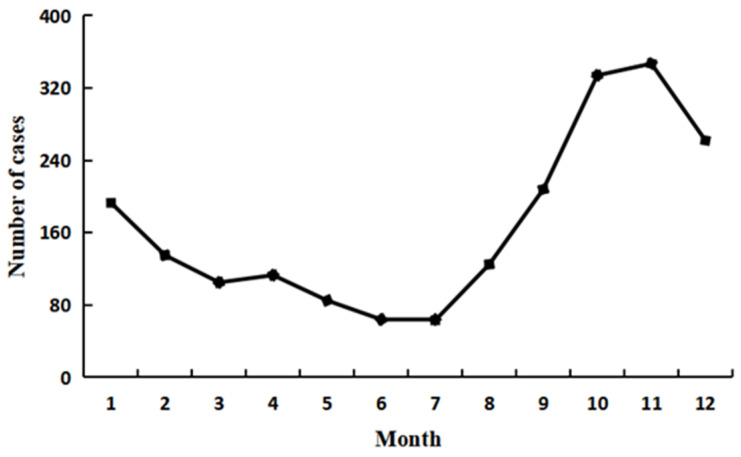
Distribution of VL by month in Xinjiang in the period 2004–2021.

**Figure 3:**
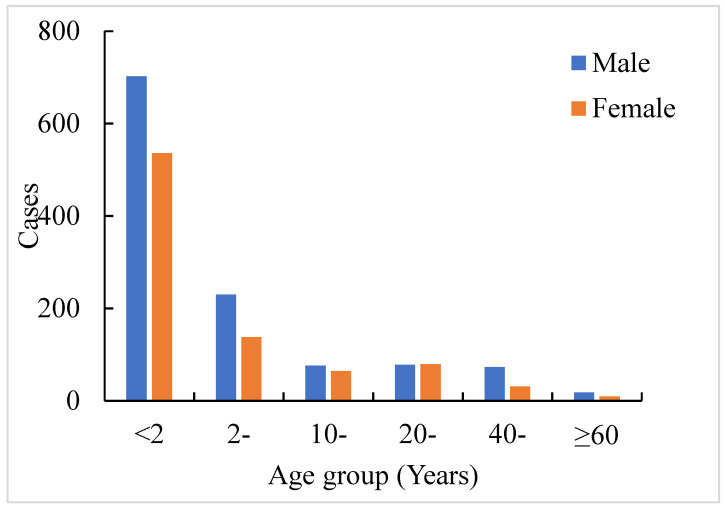
Age distribution of VL cases in Xinjiang in the period 2004–2021.

**Figure 4:**
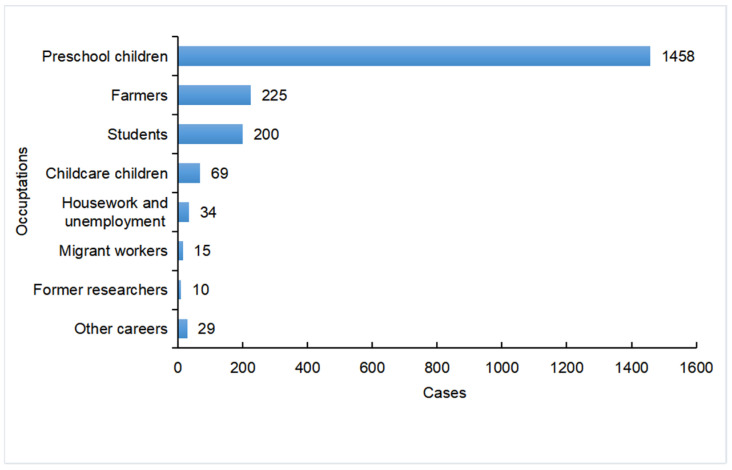
Distribution of VL in Xinjiang by occupation in the period 2004–2021.

During our study period, the incidence of VL in Xinjiang exhibited several distinctive trends. Seasonal pattern analysis showed that VL typically occurred from the peak of October to February of the next year, with this period accounting for 62.49% of all cases (Figure 2). The highest incidence was in November, accounting for 17.06% of cases, and the lowest incidence was in July, accounting for 3.09% of cases. Moreover, the highest incidence of VL was in the age group of 0–3 years, which accounted for 66.03% of cases (1347/2040), and decreased with age (Figure 3). The minimum age was 23 days and the maximum age was 77 years. The majority of the cases were reported in scattered children (71.63%, 1457/2034), followed by farmers (11.01%, 224/2034) and students (9.68%, 197/2034) (Figure 4).

The counties and cities with significantly high prevalence were predominantly located in seven regions and two autonomous prefectures in Xinjiang. In particular, Kashgar accounted for a considerably high number of cases (1688 cases, 82.98%). From 2004 to 2008, the number of cases in Aksu tended to stabilize; it gradually decreased in recent years, with 124 cases reported in total, accounting for 6.09% of all cases. Further, Hotan accounted for 1.52% (31 cases); Bayingoleng Mongol Autonomous Prefecture for 3.63% (74 cases); Hami for 0.19% (6 cases); Kizilsu Kirgiz Autonomous Prefecture for 4.08% (83 cases); and Turpan City for 0.54% (11 cases) (Table 1). The other regions reported 17 imported cases in recent years.

### 3.2. Global Spatial Autocorrelation Analysis and Local Indicators of Spatial Association of VL

**Figure 5:**
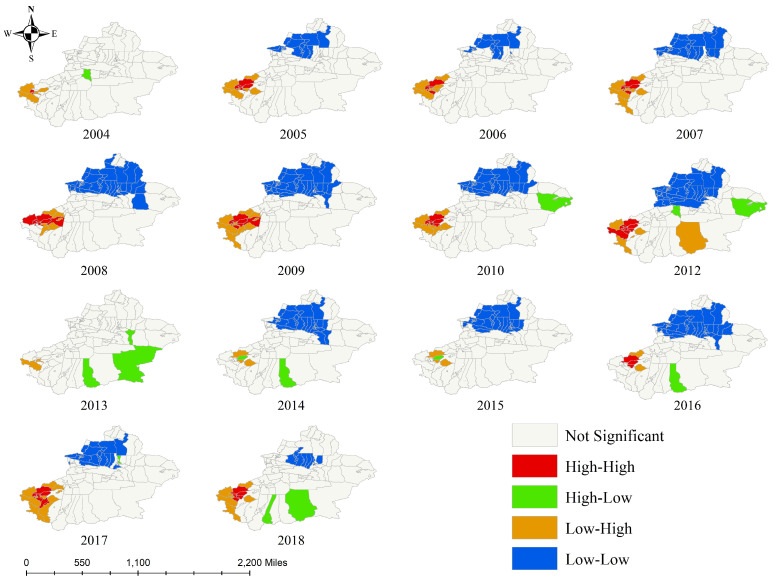
Spatial autocorrelation analysis of VL in Xinjiang in 14 representative years.

The global spatial autocorrelation analysis indicated that positive spatial clustering was present from 2004 to 2021 (Moran’s I = 0.74, Z = 4.900, *p* < 0.05) (Table 2). Moran’s scatter diagram shows the spatial clustering of counties affected by the epidemic. The results of the local spatial autocorrelation analysis are subsequently displayed in the chart of a LISA clustering graph. The “high–high” areas were characterized by significantly higher values, with clustering in Shufu County, Shule County, Jiashi County, Atushi City, Yingjisha County, Kashgar City, and Yuepuhu County. The “high–low” areas were located in Kuche City, Minfeng County, Qiemo County, Ruoqiang County, and Luntai County. In contrast, the “low–high” areas were identified in Wuqia County, Akto County, Zepu County, Aheqi County, and Maigaiti County. The “low–low” areas were located in Qitai County, Fuyun County, Emin County, Yumin County, Bole City, and Wenquan County. The aggregation showed dynamic changes in VL incidence. In general, the clustering areas were mainly located in northwestern Xinjiang from 2004 to 2021. Moreover, autocorrelation clustering at the county level in Xinjiang by year is displayed in Figure 5.

### 3.3. Spatiotemporal Clusters of VL

Scan statistics were used to detect statistically significant county-level clusters of infection. Based on the SaTScan analysis, scan statistics were also used to detect statistically significant clusters of infection at the county level. Four spatial and temporal agglomerations were identified during our study period. They were distributed over six counties and had a gathering time from 2004 to 2011. The Class I aggregation area was in Jiashi County (39.591334 N, 77.219128 E) for the period August 2008 to January 2017. The actual number of cases in this region was 1037, whereas the expected number was 18.85. The RR of VL infection in this region was very high at 111.10 (LLR = 3454.06, *p* < 0.01). The Class II aggregation area was in Kucha City and Luntai County (41.774720 N, 83.448610 E) for the period January 2005 to November 2009, with a radius of 92.93 km. The actual number of cases in this region was 55, whereas the expected number was 14.29 (RR = 3.93, LLR = 3454.06, *p* < 0.01). The Class III aggregation area was in Yuli County (40.846022 N, 86.848431 E) for the period August 2010 to January 2011. The actual number of cases in this region was 11, whereas the expected number was 0.26 (RR = 42.73, LLR = 30.532265, *p* < 0.01). The Class IV aggregation areas were in Qiemo County and Minfeng County (38.067874 N, 85.487348 E), with a radius of 216.36 km, for the period September 2012 to December 2016. The actual number of reported cases was 11, whereas the expected number was 0.26 (RR = 2.28, LLR = 8.40, *p* < 0.01) (Table 3).

## 4. Discussion

The present study is a retrospective analysis of the epidemiology of VL in Xinjiang, China, between 2004 and 2021. The findings shed light on its spatiotemporal distribution and provide important information for the development of prevention and control strategies for the future.

The temporal profile of the incidence of VL in Xinjiang during 2004 to 2021 exhibited substantial fluctuations, with peaks in 2008 and 2015. This trend was basically the countywide trend in China. As one of the most highly affected VL endemic areas in Xinjiang, China, Kashi Prefecture had two outbreaks during 2005 to 2015, which significantly affected the trend for mainland China [11]. The increased prevalence in Kashgar could be attributed primarily to its unique geography, which is characterized by the coexistence of deserts and oases. The western part of the region is predominantly occupied by green agricultural areas, whereas the eastern part is dominated by desert landscapes. Consequently, AVL and DT-AVL coexist in this region. However, in Jiashi County in the Kashi region, it was difficult to determine the distribution and species of vertebrate hosts of DT-AVL, and, thus, it is difficult to implement precise prevention and control measures in this region [21]. The number of reported cases of VL has been decreasing annually since 2006. The incidence of VL also declined dramatically in the past 3 years, with only two cases reported in 2021. The reduction could be attributed to the national program for the fight against poverty and the continuous implementation of preventive and control measures in Xinjiang, including mass screening, insecticide spraying, and health education programs, which have led to considerable improvement in the residential environment and sanitation levels.

The spatial profile of recent years showed that the reported cases of VL in Xinjiang were mainly scattered in seven regions and two autonomous prefectures and that most of the cases were distributed in the southern region, among which the Kashgar region had the highest number of cases. With regard to seasonal distribution, the main peak season was from October to February of the following year. These features are consistent with the epidemiological characteristics of VL [8]. It is speculated that the peak sand fly activity is mainly in late May and September, with a latent period of 2–6 months; this may explain the occurrence of cases in October and February of the following year. With regard to age-related distribution, the majority of affected individuals were children <2 years old, with the youngest patient being 23 days old and the oldest being 77 years of age. Further, the male-to-female ratio was 1.37:1. The highest incidence rates were found among males and children, and this is similar to the findings of other studies [22,23]. The higher incidence rates in males have been attributed to occupational issues, and the higher incidence rates in children have been ascribed to the incomplete development of their immune systems [24,25,26]. With rapid economic and social development, a growing number of men have been working outdoors compared with women, and the elderly and children are typically stationed locally at the site of work. These results indicate that more attention should be paid to specific age groups and that measures for effective control of the vector should be implemented during May and September.

Spatial analysis revealed that the aggregation of VL was predominantly in the southwest region of Xinjiang, and this is in alignment with the high-risk areas identified by spatiotemporal clustering analysis (class I). In recent years, the H-H clustering region has been primarily observed in Gashi County, Atushi City, Shufu County, Injisha County, Kashgar City, Yepuhu County, and Bachu County. The emergence of new H-H clustering areas in Shache County and Zephyr County in 2017 is a matter of obvious concern. This phenomenon might be associated with several factors. First, VL primarily spreads through gradual penetration into the surrounding areas and via short-distance transmission, leading to the formation of new outbreak areas. However, the development of transportation infrastructure and increased population mobility have expanded the range of activities of sandflies, thus exacerbating the occurrence of VL. Another problem is the increase in international tourism and the influx of immigrants from endemic countries, which have resulted in the disease spreading from endemic to nonendemic areas [27]. In a survey of cases imported from abroad from 2005 to 2010, 226 cases of VL were identified; importantly, the number of imported cases was higher mainly in October and November and constituted a serious public health problem [8]. Therefore, local authorities should assess the risk of local transmission caused by imported cases and control the further spread of VL.

Several recently discovered phenomena have made the prevention and control of VL more complicated. As a result, despite numerous efforts, there have been additional challenges to effectively control VL. First, it is crucial to prioritize the effective identification and control of sand fly species [28]. In Xinjiang, *Phlebotomus longiductus*, *P. wui*, and *P. alexandrina* are the main sand fly species in the old oasis, poplar-willow desert, and gravel desert regions, respectively [1]. However, each region does not constitute a single-medium transmission. Second, ecological and geographical factors create an environment conducive to the resurgence and potential outbreak of VL. For example, livestock pens provide vectors with shelter, sufficient organic matter for the larvae in the form of enormous amounts of manure and urine, and suitable breeding sites within the concrete and brick houses [29]. Precipitation and humidity affect the reproduction and survival of sand flies, while temperature affects the life cycle of infectivity. *Leishmania* protozoa and their sand fly vectors have a huge impact on the prevalence of VL [30,31]. To reduce the incidence of VL in Xinjiang, several measures need to be implemented. For AVL, rigorous census and infectious source retrieval methods should be employed to promptly detect and treat patients. Simultaneously, comprehensive prevention and control measures, such as indoor spraying to exterminate lacewings, should be undertaken to completely eradicate the epidemic. DT-AVL is characterized by transmission via wild sand flies, which can attack humans equally well in both outdoor and indoor environments. Hence, it is essential to improve public awareness and conduct education campaigns to prevent sand fly bites while also safeguarding infants and young children from these bites through the use of insecticide-treated tents or curtains, as well as repellents.

This study has some limitations. First, its primary focus was counties, which are not the smallest administrative units. Future research should explore finer spatial scales. Second, the data utilized in this study consist of the reported VL incidence rates, which may not accurately reflect the actual incidence rates. Therefore, the incidence may have been underestimated. Third, the *Leishmania* species causing reported VL cases were not characterized, which will affect the development and implementation of control measures against them.

## 5. Conclusions

In summary, our large-scale epidemiologic study revealed a decline in the annual prevalence of VL in Xinjiang from 2004 to 2021. Furthermore, it was found that VL primarily affected the southwestern region of Xinjiang and occurred predominantly among males and scattered children. Importantly, the main season for VL was from October to February of the following year. Therefore, to effectively prevent and control this epidemic in Xinjiang, national and local health departments should optimize information sharing, strengthen joint prevention and control measures, carry out public-health-education programs, provide rural health clinics with up-to-date equipment and facilities, and conduct regular training programs to provide people with the skills and knowledge necessary for the prevention and treatment of VL and to improve their awareness of potential high-risk areas. Lastly, further research directed at developing disease-control strategies should be conducted at the city or county level to further explore the epidemiological patterns of VL in different regions and to better guide control strategies.

## Figures and Tables

**Table 1:** Regional distribution of visceral leishmaniasis (VL) cases in the period 2004–2021.

Year	Aksu Region	Hotan Region	Kashgar Region	Bayingolin Mongolian Autonomous Prefecture	Hami	Kizil Kirgiz Autonomous Prefecture	Turpan City	Another Area	Cumulative Number of Reported Cases	Annual Mean Incidence (per 100,000 Individuals)
2004	7	0	2	0	0	0	0	1	10	0.05
2005	51	12	36	36	2	6	2	8	153	0.77
2006	21	11	31	30	4	6	5	8	116	0.58
2007	45	5	15	8	0	36	4	0	113	0.55
2008	0	0	282	0	0	25	0	0	307	1.47
2009	0	0	274	0	0	6	0	0	280	1.31
2010	0	0	129	0	0	3	0	0	132	0.61
2011	0	0	49	0	0	1	0	0	50	0.23
2012	0	0	40	0	0	0	0	0	40	0.18
2013	0	0	22	0	0	0	0	0	22	0.10
2014	0	0	155	0	0	0	0	0	155	0.68
2015	0	0	403	0	0	0	0	0	403	1.75
2016	0	0	185	0	0	0	0	0	185	0.78
2017	0	0	38	0	0	0	0	0	38	0.16
2018	0	0	15	0	0	0	0	0	15	0.06
2019	0	0	4	0	0	0	0	0	4	0.02
2020	0	1	8	0	0	0	0	0	9	0.04
2021	0	2	0	0	0	0	0	0	2	0.01
Total	124	31	1688	74	6	83	11	17	2034	0.50

**Table 2:** Moran’s spatial autocorrelation coefficient for the occurrence of VL in Xinjiang in the period 2004–2021.

Year	Moran’s I	Z	*p*-Value
2004	0.103	4.130	<0.05
2005	0.148	6.899	<0.05
2006	0.130	5.377	<0.05
2007	0.166	6.412	<0.05
2008	0.029	2.938	<0.05
2009	0.049	3.836	<0.05
2010	0.182	7.061	<0.05
2011	0.081	5.537	<0.05
2012	0.130	6.219	<0.05
2013	0.100	4.514	<0.05
2014	0.004	1.224	>0.05
2015	0.001	0.827	>0.05
2016	0.031	3.141	<0.05
2017	0.211	7.493	<0.05
2018	0.198	6.752	<0.05
2019	−0.017	−0.262	>0.05
2020	0.041	1.781	>0.05
2021	0.053	2.448	<0.05
**Average**	0.074	4.900	<0.05

**Table 3:** Results from spatiotemporal clustering analysis of VL in Xinjiang in the period 2004–2021.

Date	Gathering Classification	Number of Districts/Counties Involved	Location (County or District)	Coordinates of the Aggregation Center	Scanning Radius (km)	Actual Number of Cases	Expected Number of Cases	RR	LLR	*p*-Value
August 2008–January 2017	Level 1	1	Jiashi County	39.591334 N, 77.219128 E	0	1037	18.85	111.10	3454.068374	<0.01
January 2005–November 2009	Level 2	2	Kuqa City, Luntai County	41.774720 N, 83.448610 E	92.93	55	14.29	3.93	33.825853	<0.01
August 2010–January 2011	Level 3	1	Yuli county	40.846022 N, 86.848431 E	0	11	0.26	42.73	30.532265	<0.01
September 2012–December 2016	Level 4	2	Chimu County, Min Feng County	38.067874 N, 85.487348 E	216.36	19	2.28	8.40	23.631129	<0.01

## Data Availability

The data presented in this study are available from the corresponding author upon reasonable and justified request.
